# Spontaneous Changes in Taste Sensitivity of Single Units Recorded over Consecutive Days in the Brainstem of the Awake Rat

**DOI:** 10.1371/journal.pone.0160143

**Published:** 2016-08-01

**Authors:** Joshua D. Sammons, Michael S. Weiss, Olga D. Escanilla, Andrew F. Fooden, Jonathan D. Victor, Patricia M. Di Lorenzo

**Affiliations:** 1 Department of Psychology, Binghamton University, Binghamton, New York, United States of America; 2 Feil Family Brain and Mind Research Institute and Department of Neurology, Weill Cornell Medical College, New York, New York, United States of America; Australian National University, AUSTRALIA

## Abstract

A neuron’s sensitivity profile is fundamental to functional classification of cell types, and underlies theories of sensory coding. Here we show that gustatory neurons in the nucleus of the solitary tract (NTS) and parabrachial nucleus of the pons (PbN) of awake rats spontaneously change their tuning properties across days. Rats were surgically implanted with a chronic microwire assembly into the NTS or PbN. Following recovery, water-deprived rats had free access to a lick spout that delivered taste stimuli while cellular activity was recorded. In 12 rats for the NTS and 8 rats for the PbN, single units could be isolated at the same electrode on consecutive days (NTS, 14 units for 2–5 consecutive days, median = 2 days; PbN, 23 units for 2–7 days, median = 2.5 days). Waveforms were highly similar (waveform template correlation > 0.99) across days in 13 units in NTS and 13 units in PbN. This degree of similarity was rare (0.3% of pairs in NTS, 1.5% of pairs in PbN) when the waveforms were from presumed-different neurons (units recorded on nonconsecutive days with at least one intervening day in which there were no spikes, or from different wires or rats). Analyses of multi-day recordings that met this criterion for “same unit” showed that responses to taste stimuli appeared, disappeared, or shifted in magnitude across days, resulting in changes in tuning. These data imply, generally, that frameworks for cell classification and, specifically, that theories of taste coding, need to consider plasticity of response profiles.

## Introduction

The neural representation of sensory stimuli or events is mediated by neurons that are specialized to respond to a subset of features within the sensory domain. For example, in the gustatory system, selectivity of taste-responsive cells in the periphery and brain forms the bedrock of contemporary theories of taste coding. In particular, the “one cell—one taste” idea, i.e. a labeled line, has been advanced by compelling evidence provided by molecular biological techniques that each taste quality is represented by separate groups of dedicated taste receptors and corresponding nerve fibers [1]. While taste-responsive cells in the CNS are more broadly tuned across taste qualities than are cells in the periphery, functionally distinct cell types can be defined by the tastant that evokes the most vigorous response, called the “best” stimulus [2]. These taste-quality-specific cell types are evident as a chemotopic map in the gustatory cortex [3–5].

The stability of the complement of sensitivities of each cell is an assumption that is implicit in theories of sensory coding. However, unlike in other sensory systems, taste receptor cells turn over after their limited lifespan of 10–14 days [6], raising the possibility that taste sensitivity profiles may be labile. In the periphery, long-term recordings (up to 21 days) from single fibers in the chorda tympani nerve (mediating taste on the rostral tongue) show that their response profiles change over time [7]. Although these recordings were made from rejuvenated chorda tympani (CT) fibers, the authors hypothesized that these effects were likely due to reorganized input resulting from the turnover in taste receptor cells. However, whether taste receptor turnover leads to changes in taste sensitivity in central neurons is unknown, as there may be some central compensation that maintains the stability of taste sensitivity in the face of changing afferents.

Here, we report that in awake freely-licking rats, neurons recorded in the nucleus of the solitary tract (NTS) and the parabrachial nucleus of the pons (PbN), respectively the first and second neural relays for gustatory information in the brain, show spontaneous and significant changes in taste response profiles over consecutive days. Thus, central systems for coding and processing taste stimuli must function in the face of a dynamically changing set of tuning properties in their brainstem afferents.

## Results

Taste responses to NaCl (salty), citric acid (sour), sucrose (sweet), quinine HCl (bitter), monosodium glutamate (umami), and distilled water or artificial saliva [8] were recorded from single neurons in the NTS and PbN of awake, freely licking rats. Detailed results of the taste-responsive properties of these cells in NTS [9] and PbN [10] have been reported previously. Twelve rats for the NTS and eight rats for the PbN yielded multiday recordings. In the NTS, 79 well-isolated units were recorded; 14 units of these were recorded from the same wire for 2–5 days (median = 2 days) (“Consecutive” recordings). In the PbN, 62 units were recorded; 23 of these were Consecutive (range = 2–7 days; median = 2.5 days).

To determine whether Consecutive recordings were likely to represent recordings of the same neuron, we examined the similarity of the recorded waveform templates. As detailed in Materials and Methods, our criterion was that waveform similarity was at a level rarely encountered in the population of presumed-different pairs recorded at the same electrode (0.3% of pairs in NTS, 1.5% of pairs in PbN) but with gaps of one or more days during which no activity was recorded, or at different electrodes (“Interrupted” recordings). Despite its rarity in these Interrupted recordings, this level of waveform similarity was common (52% in NTS, 45% in PbN) in the Consecutive recordings. Consecutive recordings that met the waveform-correlation criterion were considered to arise from the same unit.

**Fig 1:**
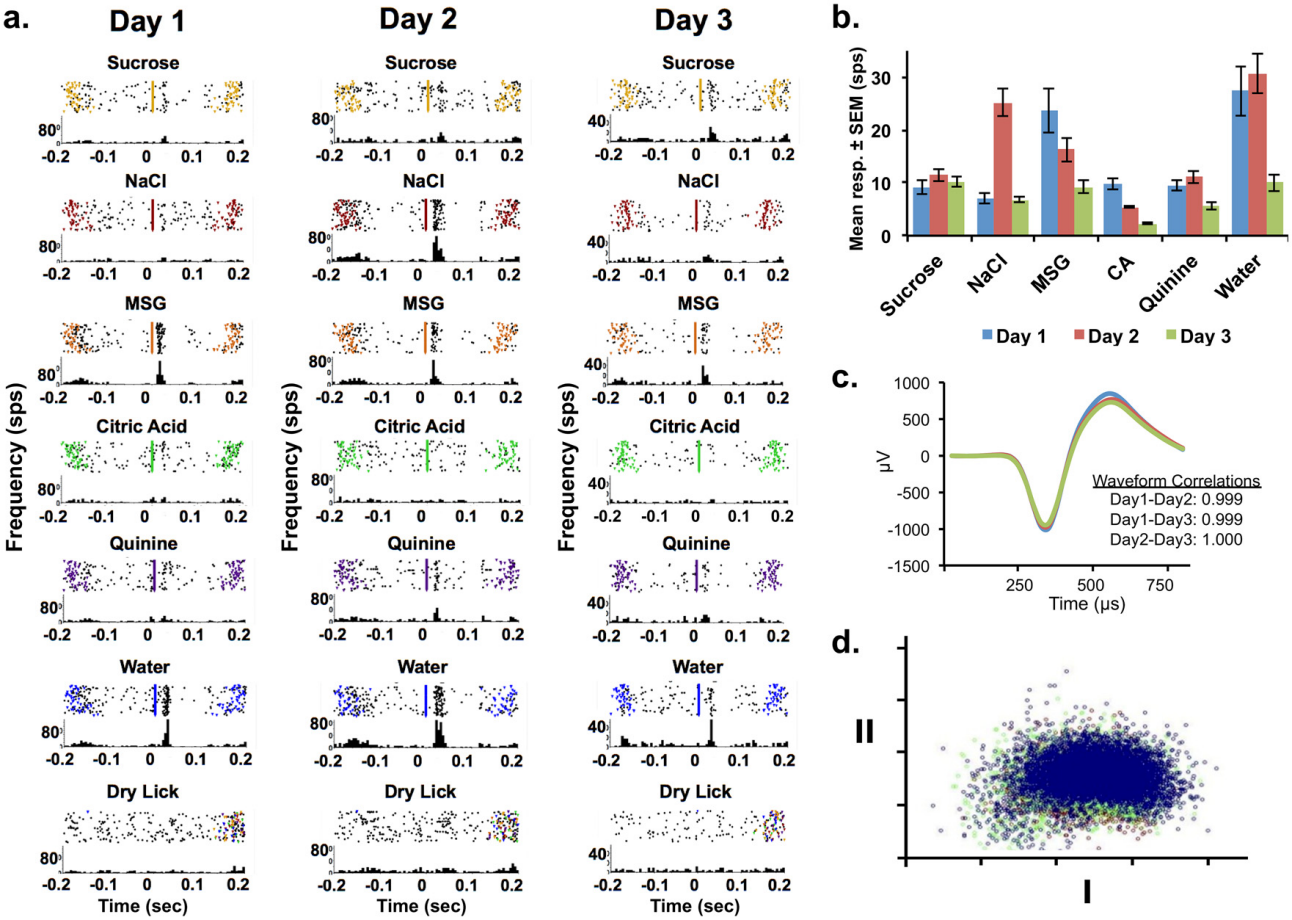
Example of a multiday recording of taste responses from a single PbN unit (#3 from Table 2). a) Rasters (top of each panel) and peristimulus-time histograms (bottom of each panel) evoked by each tastant across days. Colored triangles indicate the occurrence of a lick. b) Mean response magnitudes ± standard error of the mean (SEM) for each taste stimulus on each day. Abbreviation: CA, citric acid. This neuron showed a response to MSG that decreased across days, response to NaCl primarily on Day 2, a response to water that significantly decreased on the third day, and small responses to quinine and sucrose. c) Mean waveforms (800 μs) recorded on the three days; inset: waveform correlation between days. d) Principal components analyses results for individual waveforms recorded on each of three days, color-coded by day.

In many of the recordings of brainstem taste neurons that met the “same-unit” criterion across days, sensitivity profiles changed significantly over time (see Tables 1 and 2) in both the NTS and PbN. Considering the raw responses, 12 of 13 NTS units and 9 of 13 PbN units the best stimulus changed over days (Tables 1a and 2a). In addition, there were several cases where cells did not respond to a given tastant on one day but a response emerged on a subsequent day (e.g. NTS cells 2, 7, 12, PbN cells 4, 7, 12, 13) or vice versa (e.g. NTS cells 7, 8, PbN cells 2, 4, 10, 11). Fig 1 shows an example of this effect in a PbN cell (cell 3) recording over three days. Moreover, there were significant changes in the sizes of responses to the best stimulus (e.g. PbN cells 9, 10), and to secondary stimuli (e.g. NTS cell 7, PbN cell 1).

**Table 1:** Taste response magnitudes in spikes per sec (sps) in the NTS for taste stimuli of multiday recordings that met the “same unit” criterion.

a. Raw									b.Baseline-subtracted							
Unit	Day	S	N	CA	Q	M	W/AS	SFR	Unit	Day	S	N	CA	Q	M	W/AS
1	Day 1	30.4 NR	*23*.*9*	**44.8**	31.9 NR	28.0 NR	27.7 NR	44	1	Day 1	-1.6 NR	*-6*.*9*	**12.4**	2.8 NR	2.2 NR	0.8 NR
	Day 2	35.9 NR	*28*.*9*	**40.3**	31.6	29.7 NR	*26*.*1*	22.6		Day 2	1.2 NR	*-10*.*6*	**12.2**	8.4	-0.7 NR	*-10*.*1*
	Day 3	*23*.*9*	32.4 NR	**42.0**	28.5 NR	*23*.*0*	*24*.*3*	10.5		Day 3	*-9*.*6*	0.6 NR	**9.7**	-0.3 NR	*-11*.*5*	*-14*.*7*
2*	Day 1	23.9 NR	24.1 NR	26.2 NR	24.2 NR	23.8 NR	26.2 NR	52.3	2*	Day 1	3.1 NR	0.1 NR	5.3 NR	-0.3 NR	-3.2 NR	0.7 NR
	Day 2	26.8 NR	25.5 NR	27.9 NR	**39.8**	25.9 NR	24.8 NR	42.5		Day 2	-2.5 NR	-3.1 NR	1.0 NR	**12.1**	0.6 NR	-3.7 NR
	Day 3	**46.3**	27.9 NR	29.9 NR	29.6 NR	29.2 NR	27.6 NR	58.4		Day 3	**18.0**	0.6 NR	1.9 NR	-0.6 NR	1.4 NR	-0.4 NR
3*	Day 1	*3*.*2*	*1*.*5*	*2*.*9*	***1*.*4***	*1*.*6*	*2*.*0*	9.3	3*	Day 1	***-8*.*5***	*-7*.*5*	*-8*.*4*	*-6*.*2*	*-4*.*8*	*-7*.*3*
	Day 2	*1*.*6*	*1*.*9*	*3*.*9*	*1*.*7*	*6*.*0*	***1*.*1***	11.9		Day 2	*-6*.*9*	*-5*.*5*	*-8*.*7*	***-9*.*5***	*-7*.*0*	*-7*.*1*
	Day 3	***1*.*1***	*6*.*2*	*4*.*3*	*2*.*8*	*3*.*6*	*2*.*4*	14.4		Day 3	*-5*.*5*	*-7*.*3*	*-5*.*3*	*-6*.*1*	*-4*.*8*	***-9*.*0***
4*	Day 1	*1*.*7*	***1*.*0***	0.6 NR	*1*.*2*		9.0 NR	5.7	4*	Day 1	*-6*.*7*	***-13*.*2***	-2.0 NR	*-8*.*0*		-6.3 NR
	Day 2	*1*.*3*	*1*.*4*	***0*.*9***	*1*.*4*		8.8 NR	10.9		Day 2	*-7*.*5*	***-7*.*6***	*-7*.*3*	*-6*.*4*		-0.4 NR
	Day 3	*0*.*4*	*0*.*7*	*0*.*6*	*0*.*3*^1^		**12.7**	25		Day 3	*-6*.*4*	*-8*.*9*	***-12*.*4***	*-6*.*1*		7.9
5*	Day 1	*24*.*3*	*24*.*2*	***18*.*7***	*27*.*6*		*30*.*5*	17.7	5*	Day 1	*-76*.*3*	***-93*.*5***	*-79*.*8*	*-79*.*9*		-45.3
	Day 2	***24*.*0***	*24*.*0*	*29*.*8*	*26*.*0*		54.8^1^ NR	16.2		Day 2	***-78*.*3***	*-69*.*8*	*-62*.*5*	*-76*.*0*		-0.7^1^ NR
6*	Day 1	13.6 NR	17.2 NR	28.7	15.5 NR	**29.7**	23.7	2.5	6*	Day 1	-6.8 NR	2.2 NR	15.9	1.5 NR	**18.5**	12.9
	Day 2	**35.0**^1^	*11*.*2*	16.0 NR	*6*.*8*	15.8 NR	18.0 NR	1.1		Day 2	**18.0**	*-10*.*0*	6.0 NR	*-8*.*2*	1.8 NR	6.8 NR
7*	Day 1	18.8 NR	**42.2**	25.4 NR	36.2	22.3 NR	22.0 NR	0	7*	Day 1	-10.8 NR	**19.2**	3.4 NR	17.5	-6.4 NR	-2.8 NR
	Day 2	**34.0**	*6*.*0*^1^	18.3 NR	18.4^1^ NR	13.0^1^ NR	30.0	0		Day 2	**18.8**	*-13*.^1^	-0.5 NR	-2.8 NR	-1.7 NR	16.4
8*	Day 1	17.4	21.2	17.6	**23.7**		12.0	5	8*	Day 1	10.0	**14.2**	9.8	10.3		7.6
	Day 2	10.0 NR	8.5 NR	**13.8**	13.7		13.3	11.4		Day 2	2.2 NR	-0.5^1^ NR	**11.0**	10.9		7.7
9*	Day 1	34.4	30.7 NR	38.8	23.9 NR	**61.6**	23.4 NR	17.8	9	Day 1	15.8	1.4 NR	13.6	1.0 NR	**41.6**	-3.0 NR
	Day 2	**42.5**	35.2	22.3 NR	*17*.*7*	36.9^1^	*7*.*5*^1^	4.6		Day 2	16.8	16.2	-4.0 NR	*-13*.*5*	**19.7**^1^	*-14*.*5*
10*	Day 1	*8*.*9*	*9*.*2*	**21.3**	*9*.*6*	*11*.*8*	*11*.*1*	40.6	10*	Day 1	*-7*.*8*	*-6*.*5*	8.0	***-9*.*4***	*-6*.*9*	*-8*.*1*
	Day 2	*12*.*8*	17.9^1^ NR	*12*.*4*	*15*.*2*	*12*.*1*	***8*.*3***	37.3		Day 2	***-9*.*6***	0.1 NR	*-8*.*6*^1^	*-7*.*9*	*-8*.*1*	*-8*.*4*
11*	Day 1	*5*.*7*	*6*.*4*	*5*.*9*	*4*.*8*		**83.0**	3.5	11*	Day 1	*-30*.*9*	*-19*.*2*	*-34*.*5*	*-22*.*6*		**38.2**
	Day 2	*0*.*2*^1^	2.5^1^ NR	1.3^1^ NR	**2.4**		1.5^1^ NR	0.2		Day 2	***-3*.*6***	1.1^1^ NR	-0.7 NR	2.2^1^		0.3 NR
12*	Day 1	3.3	1.9 NR	**3.7**	2.7		0.7 NR	0.1	12*	Day 1	2.7	0.5 NR	**2.9**	1.9		-0.1 NR
	Day 2	5.4	**7.3**^1^	6.7	6.3		1.8^1^ NR	0.7		Day 2	4.2	**5.7**^1^	5.7	5.5		-1.4 NR
13*	Day 1	*0*.*5*	*0*.*3*	***0*.*2***	*0*.*4*		2.9 NR	3.8	13*	Day 1	***-3*.*3***	*-3*.*1*	*-2*.*6*	*-1*.*6*		0.1 NR
	Day 2	*5*.*0*^1^	*3*.*1*^1^	*7*.*1*^1^	*4*.*6*^1^		**28.2**^1^	13.8		Day 2	*-8*.*2*^1^	*-11*.*9*^1^	*-10*.*9*	*-13*.*0*^1^		**14.4**

^1^, significant difference from day 1;

^2^, significant difference from day 2;

*, change in best stimulus across days;

Italicized responses indicate inhibitory responses; bold numbers indicate the response to the “best” stimulus; S, sucrose; N, NaCl; CA, citric acid; Q, quinine; M, MSG; W, water; AS, artificial saliva (underlined); SFR, spontaneous firing rate.

**Table 2:** Taste response magnitudes in spikes per sec (sps) in the PbN for taste stimuli of multiday recordings that met the “same unit” criterion.

a. Raw									b.Baseline-subtracted							
Unit	Day	S	N	CA	Q	M	W/AS	SFR	Unit	Day	S	N	CA	Q	M	W/AS
1*	Day 1	48.0	**55.0**	53.3	45.2	49.7	42.6	0	1*	Day 1	28.2	**33.2**	29.5	22.1	27.3	23.9
	Day 2	39.2	24.8^1^	35.3	34.7	23.3^1^	**50.3**	0		Day 2	24.2	11.8^1^	23.8	23.3	10.3^1^	**32.5**
	Day 3	44.3	39.6^2^	35.3	30.1	40.2^2^	**51.5**	9.6		Day 3	25.7	**42.1**^2^	21.8	16.1	29.6^2^	19.4
	Day 4	43.5	**55.2**^2^	39.0	31.2	51.2^2^	34.7	0		Day 4	33.3	48.9^2^	37.4	40.0	**54.6**^2^	29.3
2*	Day 1	24.5	**35.0**	14 NR	*7*.*3*	27.5	28.0	3.2	2	Day 1	8.0	**18.4**	1.4 NR	*-7*.*5*	13.7	12.2
	Day 2	15.4 NR	**28.2**^1^	15.4 NR	*3*.*5*	27.5^1^	11.7 NR	7		Day 2	-1.4 NR	**16.2**^1^	-3.7 NR	*-8*.*7*	9.8^1^	-5.1 NR
	Day 3	16.7 NR	17.9^1^,^2^	13.8 NR	*2*.*4*	**19.2**^2^	11.7 NR	9.6		Day 3	3.5 NR	**10.1**^1^,^2^	-0.4 NR	*-7*.*4*	7.0^2^	0.7 NR
3*	Day 1	23.6	21.5	23.1	24.6	58.3	**60.8**	6.8	3*	Day 1	19.9	18.3	18.7	18.8	**56.3**	55.2
	Day 2	**22.9**	10.3	1.8^1^	13.1	5.6^1^	19.5^1^	5.9		Day 2	**18.6**	7.1	*-2*.*1*^1^	9.0	3.5^1^	15.8^1^
	Day 3	19.3	11.1	2.1^1^ NR	15.2	26.7^1^,^2^	**27.6**^1^	4.7		Day 3	17.1	9.5	*-0*.*9*^1^	11.8	**24.2**^1^,^2^	23.3^1^
4*	Day 1	16.2	11.7 NR	*4*.*6*	**16.9**	12.3 NR	11.4 NR	0.4	4*	Day 1	9.0	-1.8 NR	***-9*.*7***	9.4	-6.9 NR	-1.8 NR
	Day 2	18.8	**20.0**	8.9 NR	*6*.*0*	19.0	21.1	2.4		Day 2	**12.8**	9.5	0.1 NR	*-11*.*4*	9.0	9.3
5*	Day 1	61.3	58.9	12.3	*9*.*8*	**71.1**	22.1 NR	3.7	5	Day 1	**40.5**	25.3	*-17*.*2*	*-19*.*1*	39.1	1.3 NR
	Day 2	**51.5**^1^	45.3	33.0^1^	*8*.*6*	48.2^1^	30.0	2.1		Day 2	**31.7**	24.5	16.0^1^	*-10*.*5*	24.6^1^	11.0
6*	Day 1	29.7	27.1	26.9	**30.0**	13.2 NR	18.8	7.9	6	Day 1	**17.4**	13.1	9.9	15.3	-6.3 NR	10.0
	Day 2	**14.5**^1^	10.1^1^	10.9^1^	12.5^1^	9.7	12.7	3.7		Day 2	**8.2**	7.8^1^	5.6^1^	8.0	5.5	8.0
7*	Day 1	33.3	25.7	3.4 NR	28.3	21.3	**53.2**	9.1	7*	Day 1	23.2	27.5	-1.0 NR	23.7	17.3	**48.8**
	Day 2	17.3	19.6	18.7^1^	**32.8**	25.8	25.8^1^	5.1		Day 2	17.1	13.8	14.7^1^	**30.8**	24.3	22.3^1^
8	Day 1	23.6 NR	**26.5**	22.6 NR	19.9 NR	29.7	19.4 NR	2.9	8*	Day 1	2.1 NR	9.9	1.7 NR	0.2 NR	**12.4**	1.2 NR
	Day 2	25.5	**25.9**	18.6 NR	22.3	18.5^1^ NR	23.2	3.1		Day 2	**11.9**^1^	9.6	1.3 NR	8.8	-2.2^1^ NR	8.2
9	Day 1	**17.6**	16.9	8.5 NR	17.3	7.4 NR	10.4	7.9	9	Day 1	**9.6**	9.6	-0.3 NR	9.5	-3.4 NR	6.1
	Day 2	**10.1**^1^	7.0^1^	8.2	5.5^1^	7.3	8.8	3.7		Day 2	**6.1**	5.7^1^	4.9	3.0	4.0	5.3
10	Day 1	16.6	14.8 NR	**26.0**	25.5	17.1	12.3 NR	18.1	10*	Day 1	8.5	1.9 NR	15.8	**16.7**	6.7	0.9 NR
	Day 2	6.5^1^ NR	8.4^1^	**12.7**^1^	12.5^1^	5.7^1^ NR	6.5^1^ NR	10.9^1^		Day 2	2.4^1^ NR	4.1^1^	**8.1**^1^	7.1^1^	1.1^1^ NR	0.9 NR
11	Day 1	**13.4**	10.7	8.0 NR	5.1 NR	11.5	10.1	3.3	11*	Day 1	7.2	6.5	2.7 NR	-2.9 NR	**7.3**	5.9
	Day 2	**8.8**	8.6	4.7^1^ NR	6.5	4.2^1^ NR	2.9^1^ NR	3.5		Day 2	**6.0**	5.9	1.5^1^ NR	3.9	-1.5^1^ NR	0.4^1^ NR
12*	Day 1	9.7 NR	**17.7**	17.1	15.4 NR	9.9 NR	10.6 NR	35.9	12*	Day 1	-3.8 NR	**12.0**	10.5	1.0 NR	1.0 NR	1.3 NR
	Day 2	5.1 NR	14.7	9.1	14.8	**15.9**	3.4 NR	44.4		Day 2	0.9 NR	9.7	5.5	10.8	**11.7**	-1.6 NR
13*	Day 1	12.5	9.1 NR	8.1 NR	**14.5**	*6*.*8*	7.8 NR	0.4	13*	Day 1	5.6	-3.8 NR	-2.1 NR	8.7	***-10*.*9***	-1.9 NR
	Day 2	14.6	15.1	7.7 NR	*4*.*8*	**15.4**	18.9^1^	2.4		Day 2	**10.3**	7.4	-1.2 NR	*-9*.*0*	5.9	8.2^1^

^1^, significant difference from day 1;

^2^, significant difference from day 2;

*, change in best stimulus across days;

Italicized responses indicate inhibitory responses; bold numbers indicate the response to the “best” stimulus; S, sucrose; N, NaCl; CA, citric acid; Q, quinine; M, MSG; W, water; AS, artificial saliva (underlined); SFR, spontaneous firing rate.

These changes could not be explained on the basis of changes in the spontaneous firing rate or the pre-stimulus baseline for the individual tastants. With regard to spontaneous firing rate (Tables 1a and 2a), only one neuron showed a significant change (PbN cell 10), but the degree of change of spontaneous firing could not account for the change in response magnitude over days. With regard to changes in baseline activity, this also could not account for our findings. Baseline firing rates (mean firing rate during the 0.5 s prior to taste stimulation) are shown in Table 3. There were four NTS and five PbN cells that showed a significant change in baseline firing rate for at least one stimulus across days. However, these changes could not account for the significant changes in the baseline-subtracted or raw changes across days. With responses recalculated after subtracting baseline activity, day-to-day changes in response profile were still present in 21 of the 26 units. Moreover, if one uses the pre-stimulus baseline-subtracted responses as the response measure, then 3 units that appeared stable (without this subtraction) then show significant day-to-day variability in their best stimulus (PbN cells 8, 10, 11).

**Table 3:** Pre-stimulus baseline firing rates in spikes per sec (sps) in the NTS and PbN for taste stimuli of multiday recordings that met the “same unit” criterion.

a. NTS								b. PbN							
Unit	Day	S	N	CA	Q	M	W/AS	Unit	Day	S	N	CA	Q	M	W/AS
1	Day 1	32.0	30.7	32.4	29.1	25.8	26.9	1	Day 1	19.8	21.8	23.8	23.1	22.4	18.7
	Day 2	34.8	39.5	28.1	23.2	30.4	36.2		Day 2	15.0	13.0	11.5	11.3	13.0	17.8
	Day 3	33.5	31.8	32.3	28.7	34.5	39.0		Day 3	17.8	13.1	17.2	15.1	21.6	15.3
									Day 4	14.8	16.7	10.6	15.3	17.6	19.6
2	Day 1	20.9	24.0	20.9	24.5	27.0	25.5	2	Day 1	16.5	16.6	12.5	14.7	13.8	15.8
	Day 2	29.3	28.7	26.9	27.8	25.3	28.5		Day 2	16.8	12.0	19.1	12.3	17.7	16.9
	Day 3	28.3	27.3	28.0^1^	30.2	27.8	28.0		Day 3	13.2	7.8	14.2	9.8	12.2	11.0
3	Day 1	11.6	9.0	11.3	7.5	6.4	9.3	3	Day 1	3.7	3.3	4.4	5.8	2.1	5.6
	Day 2	8.6	7.4	12.6	11.2	13.1	8.3		Day 2	4.3	3.2	3.9	4.1	2.1	3.7
	Day 3	6.7	13.5	9.6	8.8	8.4	11.4		Day 3	2.1	1.5	3.0	3.4	2.5	4.3
4	Day 1	8.4	14.2	2.6	9.2		15.2	4	Day 1	7.1	13.4	14.3	7.5	19.1	13.1
	Day 2	8.8	9.0	8.2	7.8		9.2		Day 2	6.0	10.5	8.9	17.4	10.0	11.8
	Day 3	6.8	9.6	13.0	6.4		4.8								
5	Day 1	100.5	117.8	98.5	107.5		75.8	5	Day 1	20.8	33.6	29.4	28.9	32.0	20.8
	Day 2	102.3	93.8	92.3	102.0		55.5		Day 2	19.8	20.8	16.9	19.1	23.6	19.0
6	Day 1	20.4	15.0	12.8	14.0	11.2	10.8	6	Day 1	12.3	14.0	17.0	14.8	19.5	8.8
	Day 2	17.0	21.2	10.0	15.0	14.0	11.2		Day 2	6.4	2.3^1^	5.3^1^	4.5	4.2^1^	4.7
7	Day 1	29.6	23.0	22.0	18.7	28.7	24.8	7	Day 1	5.8	2.5	4.3	4.7	4.0	4.3
	Day 2	15.2	19.6	18.8	21.2	14.7	13.6		Day 2	3.5	2.5	4.0	2.0	1.5	3.5^1^
8	Day 1	7.4	7.0	7.8	13.4		4.4	8	Day 1	21.6	16.6	20.9	19.8	17.3	18.2
	Day 2	7.8	9.0	2.8	2.8^1^		5.6		Day 2	13.7	16.3	17.3	13.5	20.7^1^	15.0
9	Day 1	18.7	29.3	25.2	22.9	20.0	26.4	9	Day 1	8.0	7.3	8.8	7.8	10.8	4.3
	Day 2	25.8	19.0	26.3	31.3	17.1	22.0		Day 2	4.0	1.3	3.3^1^	2.5	3.3^1^	3.5
10	Day 1	16.7	15.7	13.4	19.0	18.7	19.2	10	Day 1	8.1	12.9	10.2	8.9	10.4	11.4
	Day 2	22.4	17.8	21.0	23.2	20.3	16.8		Day 2	4.1	4.3^1^	4.7^1^	5.5	4.6^1^	5.6
11	Day 1	36.6	25.6	40.4	27.4		44.8	11	Day 1	6.2	4.2	5.3	8.0	4.2	4.2
	Day 2	3.8^1^	1.4^1^	2.0	0.2^1^		1.2		Day 2	2.8	2.7	3.2	2.7	5.7	2.5
12	Day 1	0.6	1.4	0.8	0.8		0.8	12	Day 1	13.5	5.7	6.7	14.3	8.8	9.3
	Day 2	1.2	1.6	1.0	0.8		3.2		Day 2	4.2	5.0	3.6	4.0	4.2	5.0
13	Day 1	3.8	3.4	2.8	2.0		2.8	13	Day 1	6.9	12.9	10.3	5.8	17.7	9.7
	Day 2	13.2^1^	15.0^1^	18.0^1^	17.6^1^		13.8^1^		Day 2	4.3	7.8	8.9	13.7	9.5	10.7

^1^, significant difference from day 1;

^2^, significant difference from day 2;

S, sucrose; N, NaCl; CA, citric acid; Q, quinine; M, MSG; W, water; AS, artificial saliva (underlined); SFR, spontaneous firing rate

Changes in lick pattern also are unlikely to be responsible for our findings, as we saw no significant changes in lick pattern across days in the great majority of cases. There were two exceptions: in NTS cell 1, significantly different lick rates were apparent in response to citric acid on days 1 and 2; in NTS cell 2 lick rate for quinine was significant different on days 1 vs. 3. However in both cases there were no significant changes in response magnitudes. In sum, lick rate in response to taste stimuli could not account for differences in response magnitude across days.

## Discussion

Present data show that the potential for plasticity of response profiles in the gustatory system, established in the periphery [7], extends centrally to brainstem taste nuclei, NTS and PbN. That is, taste response magnitude was shown to change in some cells and to appear and disappear in others independent of licking behavior. In most cases these changes altered the tuning profile. Changes in response tuning were spontaneous and substantial, resulting in changes in the best stimulus classification of some cells. Thus, the spontaneous changes in taste profiles across days, documented here, challenge the existing coding theories that rely on taste response profiles as a defining and enduring characteristic of a cell. Together with similar evidence of taste response plasticity in peripheral nerves [7], present data suggest that relative sensitivity across taste qualities may be a changeable characteristic of a cell.

The CT single fiber recordings reported by Shimatani et al. [7] were made from regenerated nerve fibers following a nerve cut, a factor that could not be ruled out as a contributor to day-to-day variability in response profiles of single CT fibers. However, in an earlier study, Shimatani et al. [11] showed that whole nerve recordings from the CT nerve in alert rats using similar technology were stable in response magnitude over more than 3 months. They suggested that changes in response profiles of single nerve fibers might be a local phenomenon and that variability in response magnitude across fibers would be summated in whole nerve recordings. Thus, the across fiber pattern would be stable while individual fibers might be more changeable. While this hypothesis remains viable, our data show that individual neurons in the gustatory brainstem also have changeable tuning properties.

A minority of cells showed no change in taste responses across time, raising the possibility that there is a subset of cells whose taste sensitivities are immutable. However, it is also possible that taste profiles for tastants not tested here or even taste mixtures or taste-odorant mixtures may have changed across days. In addition, it is possible that these seemingly stable cells may shift response sensitivity on a slower timescale than others—or, simply that the period in which we recorded from these cells did not include days in which a sufficient number of taste receptors turned over.

Changes in taste response profiles—including changes in the best stimulus—have previously been reported in the NTS [12,13], but the changes described here are of a novel and distinct character. For example, adaptation to a given taste stimulus can alter the responses to tastants of other qualities, resulting in changes in responses profiles of NTS cells [12]. Further, recent taste history, in the form of a 100 ms taste pulse delivered to the tongue, has been shown to alter taste response profiles of about half of NTS cells [13]. This phenomenon and the changes we describe here are likely independent, as the present findings do not appear to be related to adaptation or stimulus history. The changes described in Di Lorenzo et al. [13] were induced by a stimulus, were maximal within a limited time span of a few seconds, and then revert. In contrast, the changes described here are spontaneous and occur over days.

It is reasonable to ask how a taste stimulus could evoke the same sensation across days if the neurons that encode that stimulus change their sensitivity, as present data suggest. However, there is no *a priori* reason why the same neurons need to respond in the same way to a given stimulus throughout their lifespan. In fact, one can imagine that a target neuron, for example in a higher order taste-related structure, might be receiving input from several hundred or even thousands of neurons; the impact of any one neuron would presumably be quite small. One need only assume that the preponderance and relative strengths of taste-specific inputs would drive activity in the target as well as determine its profile of taste sensitivity. Thus, afferent neurons might change their sensitivities over time, perhaps reflecting taste receptor turnover, but the population response would remain stable.

In all, given the evidently labile nature of the taste response, we suggest that the definition of cell types in the gustatory system should include an assessment of how and whether a given cell’s selectivity changes, both in response to preceding taste stimuli [13] and, as we show here, spontaneously with the passage of time.

## Materials and Methods

### Subjects

Twenty (n = 12 for NTS and n = 8 for PbN) male Sprague-Dawley rats weighing 300–450 grams were used. Rats were kept on a 12–12 hour light/dark cycle, (lights on at 0500). All animals remained healthy throughout the experiment. Food was available *ad libitum* and water was available for at least 1 hr daily. All procedures were approved by the Binghamton University Institutional Animal Care and Use Committee.

### Electrode Implantation Surgery

Rats were surgically implanted with a chronic 8-microwire assembly into the NTS or PbN following anesthesia with a ketamine (100 mg/kg, i.p.) and xylazine (14 mg/kg, i.p.) mixture. The rat’s head was shaved and prepared by wiping the scalp with betadine and alcohol. An incision was made in the scalp and the fascia was retracted. Six skull screws were implanted in the skull. The microwire assembly was lowered into the NTS or PbN under electrophysiological guidance through a hole drilled in the skull. Coordinates were: for the NTS, AP 15–15.5 mm posterior to bregma, 1.6–2.0 mm lateral to lambda; for the PbN, AP 12–12.5 mm posterior to bregma, 1.6–2.0 mm lateral to lambda. Spike activity was checked for the presence of a taste response by periodically bathing the tongue with a wash of 0.1 M NaCl. A stainless steel wire from the microwire assembly was wrapped around a skull screw to provide an electrical ground. Once a taste response was identified, the entire assembly was cemented to the skull with dental acrylic. The animal was then placed in its home cage on top of a warmed surface until it was spontaneously mobile.

During recovery, rats were given 0.05 mg of Buprenorphine HCl (s.c.) and Gentamicin (0.05 mg; s.c.) once a day for three days. Additionally, topical antibiotic (Neosporin) was applied around the head-cap once a day for five days to prevent infection. DietGel 76A (ClearH_2_O, Portland, ME) was placed in the animals’ cages to encourage eating after the surgery. Body weight and general wellbeing (gait, respiration, activity, grooming, etc.) were monitored daily. Testing began five days after surgery or when the animal attained 90% of its pre-surgical body weight.

### Taste stimuli

Taste stimuli were NaCl (0.1M; N), citric acid (0.01M; CA), sucrose (0.1M; S), quinine HCl (0.0001M; Q), monosodium glutamate (0.1M; MSG) and water (W) or artificial saliva (0.015 M NaCl, 0.022 M KCl, 0.003 M CaCl_2_ 0.0006 M MgCl_2_, pH 5.8 +/- 0.2; AS; [8]. In previous experiments [9,10], water often produced a response in the brainstem of awake rats. As a result, in subsequent experiments, we switched our rinse control stimulus to artificial saliva. However, since the formula for artificial saliva is imperfect (it is based on monkey saliva) it may contain some elements that occasionally produce responses in taste-responsive cells.

### Recording

Following recovery, rats were water-deprived for 22 hrs and placed in a testing chamber containing a lick spout. This spout sheathed separate 20 ga. stainless steel tubes used to deliver tastants and water or AS. Delivery of tastants and rinses (W or AS) were contingent upon the animal’s licking. Each “reinforced” lick (a lick that resulted in fluid delivery) delivered ~12 μl of fluid from pressurized reservoirs routed through computer-controlled solenoids. This apparatus was calibrated daily. Blocks of taste stimuli were presented in randomized order. A stimulus block consisted of five consecutive reinforced licks, with the same tastant delivered with each lick. Five W or AS licks followed, presented on a variable ratio 5 schedule. That is, W or AS licks were interspersed with 3–5 “dry licks” in which no liquid was delivered. Electrophysiological activity was monitored as the animal licked, and the precise timing of tastant deliveries, licks and spikes was recorded (S1 and S2 Files). Detailed results of the taste-responsive properties of single cells in NTS [9] and PbN [10] have been reported previously.

### Data analyses

Spontaneous rate for each cell on each day was calculated as the mean firing rate ± standard deviation (SD) in the first 10 s period without licking, measured in 1 s time bins.

As was done in prior studies [9,10], the presence or absence of a taste response was calculated using the peristimulus-time histogram (PSTH) for each stimulus. A significant response was characterized as a >2 SDs change from baseline (calculated as the mean firing rate during the 0.5 s prior to the first stimulus lick) for at least 300 ms measured in 100 ms time bins with a 20 ms moving window.

To determine taste response magnitude, it is standard practice to subtract the baseline firing rate from the firing rate evoked by a given taste stimulus. Here, we used both the “raw” response rates (Tables 1a and 2a), calculated without subtracting baseline or spontaneous firing rates, and the “baseline-subtracted” response rates (Tables 1b and 2b) calculated by subtracting the baseline firing rate for each trial from the response rate for each trial, to compare taste responses across days. We included the raw response rates because pre-stimulus firing rates are themselves variable across trials and days (see Table 3 for baseline firing rates and Tables 1a and 2a for spontaneous firing rates) and might therefore introduce a potential confound. We note that the routine practice of subtracting the pre-stimulus firing rate to determine taste response magnitude amounts to an implicit assumption that the firing rate of a cell during taste stimulation results from additive combination of stimulus-independent firing (“noise”), and taste-evoked spikes (“signal”). The target cell of this activity would then estimate the signal by subtracting the noise from the total activity. As there is no clear evidence to support or refute this assumption, we carried out all analyses both on the raw responses (raw, Tables 1a and 2a) and with baseline rates subtracted (baseline-subtracted, Tables 1b and 2b).

For both the raw and baseline-subtracted responses, taste responses across days were compared using the mean firing rate ± SD across trials for each taste response. In cases where there was no significant response to a given tastant on a given day (as determined by analysis of the PSTH), the mean firing rate ± SD across the 4 s following the first lick was calculated. A two-tailed unpaired *t*-test was used to compare firing rates, using the individual spike counts for each presentation of a tastant as the sets to be compared across days. To correct for multiple comparisons, we used a Bonferroni correction for alpha based on the number of pairwise comparisons. We indicate whether when there was not a significant taste response with respect to baseline firing rate (NR) in Tables 1 and 2. Baseline firing rates are reported in Table 3. The “best stimulus” for each cell was defined as the stimulus that evoked the largest response magnitude among those stimuli that evoked significant responses. For those cells that showed inhibitory responses to some or all stimuli, the lowest mean response represented the most effective stimulus and was thus classifies as the best stimulus.

To ensure that differences in taste-evoked firing rates were not due to differences in licking behavior, the mean number of licks ±SD across trials during the 2 s beginning with the first stimulus lick was calculated for each stimulus for each day. To compare lick responses across days, we used a two-tailed *t*-test with alpha equal to the Bonferroni correction, as in the procedure used to compare taste responses across days.

**Fig 2:**
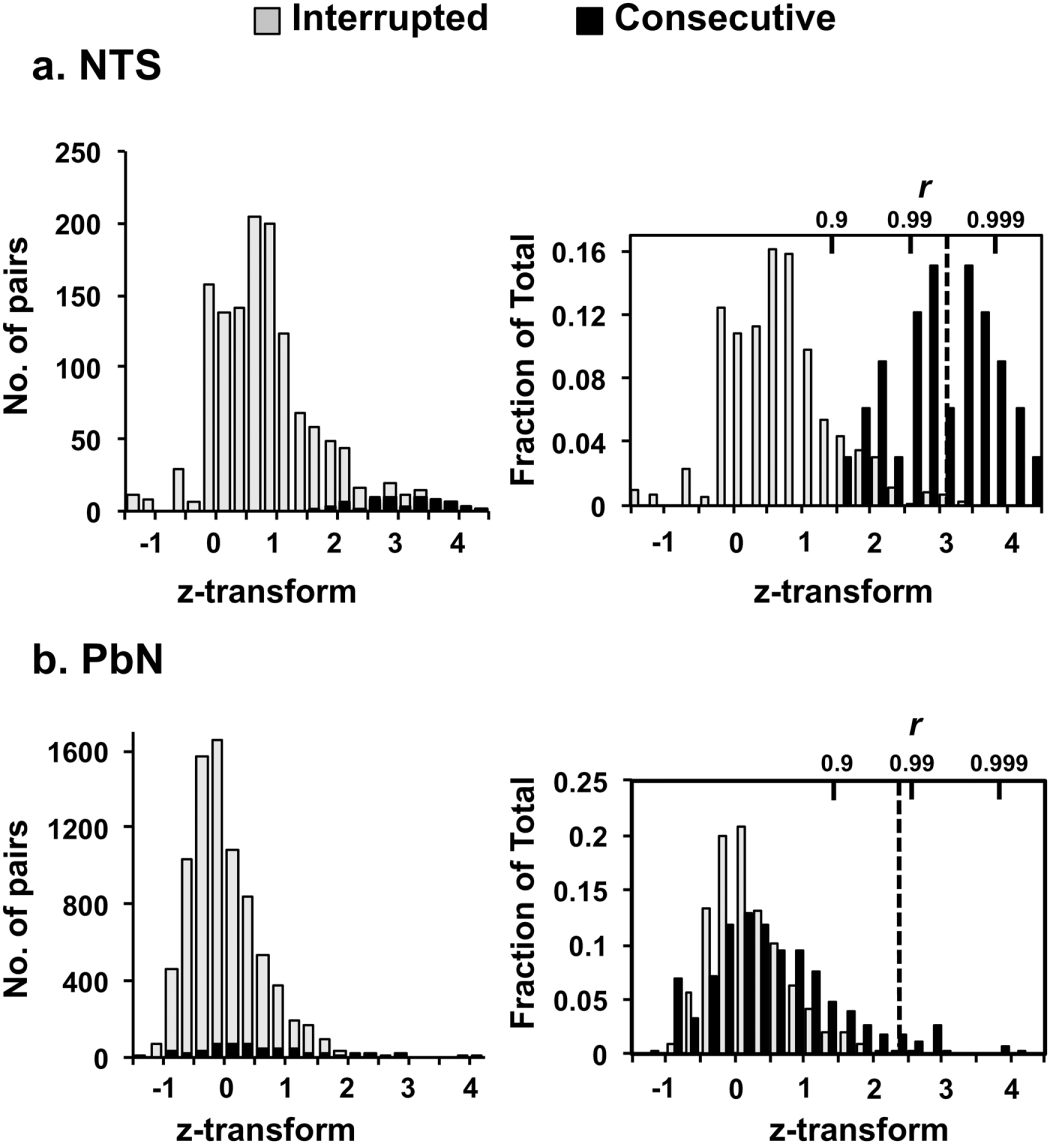
Correlation coefficients and Fisher z-transforms for all pairs of waveforms in the NTS and PBN. Large values indicate greater similarity of waveforms. Left panels in Fig 2a. for NTS and 2b. for PbN show the distributions of correlations for all pairs of waveforms. Right panels for Figures 2a. For NTS and 2b. for PbN show the same distributions but the proportion of the total in each category (Consecutive and Interrupted) is calculated for each group separately. The Consecutive group (black) consists of pairs of waveforms from units recorded from the same electrode across sequential days. The Interrupted group (presumed-different neurons; gray) consists of pairs of waveforms from units recorded on non-consecutive days at the same electrode, with at least one day of no activity intervening, or from different electrodes (including different animals), but only from animals that had multi-day recordings. The dashed vertical line in the right hand panels indicates the +2.58SD limit for z-transform values (for NTS, z = 3.04; for PbN, z = 2.47). The correspondence of z-transformed values to correlation coefficients is shown along the top of each plot on the right.

We analyzed extracellular action potential recordings from the NTS and PbN to determine whether sequential recordings on the same wire were likely to originate from the same cell. As a first step, average waveforms over 800 μs (sampled at 40kHz) were calculated for each cell on each recording session. We then calculated correlation coefficients between all pairs of waveforms (S3 File) recorded from rats with multi-day recordings. These correlations formed a skewed distribution; a small number of pairs had highly similar waveforms (Fig 2, left). To further analyze this, we classified waveform pairs into two groups: “Consecutive” recordings were from units recorded on consecutive days at the same electrode; “Interrupted” recordings, which accounted for the majority, were from units recorded at the same electrode on nonconsecutive days, with at least one intervening day in which there were no spikes, or from different electrodes, including recordings from different rats. As seen in Fig 2, waveform correlations in the Consecutive and Interrupted pairs had different distributions (two-sample Kolmogorov-Smirnov test: *p*<0.01 in NTS, *p*< 0.05 in PbN). This difference is more evident when the data were replotted with two distributions separately normalized (Fig 2, right): in the Consecutive subset, correlation coefficients were on average higher than in the Interrupted subset, and contributed disproportionately to the skewness of the combined distribution.

Interrupted pairs were presumed to be derived from different units, while Consecutive pairs could in principle either be from the same unit or different units. To identify subsets of Consecutive waveform pairs that were highly unlikely to be from different units, we further selected the Consecutive pairs according to a criterion level of waveform correlation that was rarely encountered within the Interrupted subpopulation. This criterion level, the dashed lines in the right panels of Fig 2, was determined by fitting the Fisher z-transform of all correlation coefficients to a Gaussian (since the Fisher transform approximately normalizes a distribution of correlation coefficients), and identifying the value at + 2.58 SDs, the 99^th^ percentile for a Gaussian distribution. Note that this criterion is more stringent than just using the Interrupted recordings as the basis for the comparison, as it takes into account the possibility that some of the Consecutive pairs may actually come from different units. 52% of Consecutive NTS recordings and 45% of the Consecutive PbN recordings passed this criterion; the average waveform correlations for these pairs was >0.99. In contrast, this degree of similarity was rare (0.3% of pairs in NTS, 1.5% of pairs in PbN) in the Interrupted subset. Thus, it is likely that nearly all of the Consecutive recordings above the z-transform threshold represented recordings of the same neuron.

In some cases, the waveforms drifted in shape over days such that the correlation between the waveform of the cell on the first day with the waveform of the cell on the third, fourth or fifth day no longer met the z-threshold criterion. In those cases, data from those latter days were excluded.

## Supporting Information

S1 File Timestamps for all events and spikes for each NTS neuron that met the “same unit” criterion. Datasets for each neuron that met the “same unit” criterion are separated by tabs and listed by the number in which the neuron appears in Table 1. Consecutive recording days are listed together. Timestamps for spiking activity of each NTS neuron and each event associated with that neuron are separated into columns. Timestamps for each neuron and event are listed chronologically in descending order.
(XLSX)

S2 File Timestamps for all events and spikes for each PbN neuron that met the “same unit” criterion. Datasets for each neuron that met the “same unit” criterion are separated by tabs and listed by the number in which the neuron appears in Table 2. Consecutive recording days are listed together. Timestamps for spiking activity of each PbN neuron and each event associated with that neuron are separated into columns. Timestamps for each neuron and event are listed chronologically in descending order.
(XLSX)

S3 File Waveform templates in the NTS and PbN for neurons of multiday recordings that met and did not meet the “same unit” criterion. Waveform templates for all neurons used to calculate which neurons met the “same unit” criterion. Each neuron is separated into columns with consecutive days placed adjacent to each other. Neurons that met “same unit” criterion are indicated with the cell number (in red) in which they appear in Table 1 (NTS) or Table 2 (PbN). Each waveform is broken into 32 points evenly distributed through an 800 ms window in chronological descending order.
(XLSX)

## References

[1] Yarmolinsky DA, Zuker CS, Ryba NJP. Common Sense about Taste: From Mammals to Insects. Cell. 2009;139: 234–244. 10.1016/j.cell.2009.10.001 19837029 PMC3936514

[2] Spector AC. The Representation of Taste Quality in the Mammalian Nervous System. Behavioral and Cognitive Neuroscience Reviews. 2005;4: 143–191. 10.1177/1534582305280031 16510892

[3] Accolla R, Bathellier B, Petersen CCH, Carleton A. Differential Spatial Representation of Taste Modalities in the Rat Gustatory Cortex. Journal of Neuroscience. 2007;27: 1396–1404. 10.1523/JNEUROSCI.5188-06.2007 17287514 PMC6673570

[4] Chen X, Gabitto M, Peng Y, Ryba NJP, Zuker CS. A Gustotopic Map of Taste Qualities in the Mammalian Brain. Science. 2011;333: 1262–1266. 10.1126/science.1204076 21885776 PMC3523322

[5] Sugita M, Shiba Y. Genetic tracing shows segregation of taste neuronal circuitries for bitter and sweet. Science. 2005;309: 781–785. 16051799 10.1126/science.1110787

[6] Beidler LM, Smallman RL. Renewal of cells within taste buds. J Cell Biol. The Rockefeller University Press; 1965;27: 263–272. 10.1083/jcb.27.2.263 PMC2106718 5884625

[7] Shimatani Y, Nikles SA, Najafi K, Bradley RM. Long-term recordings from afferent taste fibers. Physiology & Behavior. 2003;80: 309–315. 14637230 10.1016/j.physbeh.2003.08.009

[8] Hirata S-I, Nakamura T, Ifuku H, Ogawa H. Gustatory coding in the precentral extension of area 3 in Japanese macaque monkeys; comparison with area G. Exp Brain Res. 2005;165: 435–446. 10.1007/s00221-005-2321-y 15942736

[9] Roussin AT, D'Agostino AE, Fooden AM, Victor JD, Di Lorenzo PM. Taste Coding in the Nucleus of the Solitary Tract of the Awake, Freely Licking Rat. Journal of Neuroscience. 2012;32: 10494–10506. 10.1523/JNEUROSCI.1856-12.2012 22855799 PMC3427930

[10] Weiss MS, Victor JD, Di Lorenzo PM. Taste coding in the parabrachial nucleus of the pons in awake, freely licking rats and comparison with the nucleus of the solitary tract. Journal of Neurophysiology. American Physiological Society; 2014;111: 1655–1670. 10.1152/jn.00643.2013 PMC4035774 24381029

[11] Shimatani YY, Grabauskiene SS, Bradley RMR. Long-term recording from the chorda tympani nerve in rats. Physiology & Behavior. 2002;76: 143–149. 10.1016/S0031-9384(02)00684-4 12175597

[12] Di Lorenzo PM, Lemon CH. The neural code for taste in the nucleus of the solitary tract of the rat: effects of adaptation. Brain Research. 2000;852: 383–397. 10.1016/S0006-8993(99)02187-3 10678766

[13] Di Lorenzo PM, Lemon CH, Reich CG. Dynamic coding of taste stimuli in the brainstem: effects of brief pulses of taste stimuli on subsequent taste responses. Journal of Neuroscience. 2003;23: 8893–8902. 14523091 10.1523/JNEUROSCI.23-26-08893.2003 PMC6740402

